# Temporal changes and gender differences related to orofacial symptoms in two cohorts of 75‐year‐old Swedish subjects examined in 2007 and 2017: A repeated cross‐sectional study

**DOI:** 10.1002/cre2.671

**Published:** 2022-10-03

**Authors:** Anders Johansson, Ridwaan Omar, Josefin Sannevik, Berit Mastrovito, Gunnar E. Carlsson, Ann‐Katrin Johansson

**Affiliations:** ^1^ Department of Clinical Dentistry—Prosthodontics, Faculty of Medicine University of Bergen Bergen Norway; ^2^ Department of Restorative Sciences—Prosthodontics, Faculty of Dentistry Kuwait University Safat Kuwait; ^3^ Department of Dentistry Örebro County Council Örebro Sweden; ^4^ Dental Commissioning Unit Östergötland County Council Linköping Sweden; ^5^ Department of Prosthetic Dentistry The Sahlgrenska Academy at Göteborg University Göteborg Sweden; ^6^ Department of Clinical Dentistry—Cariology, Faculty of Medicine University of Bergen Bergen Norway

**Keywords:** aged, gender equity, mouth diseases, population

## Abstract

**Objectives:**

To compare two cohorts of 75‐year‐old persons, born 10 years apart, in regard to reported symptoms related to temporomandibular disorders (TMD) and orofacial complaints with special reference to gender differences.

**Material and Methods:**

In 2007, a questionnaire comprising questions on social factors, general and oral health, and a series of attitude‐related questions was mailed to all individuals born in 1932 living in two Swedish counties (*N* = 5195), and in 2017 to all born in 1942 (*N* = 7204). The response rate for the cohort examined in 2007 was 71.9% (*n* = 3735) and 70.7% (*n* = 5091) for the cohort examined in 2017. Bivariate statistical analyses were applied.

**Results:**

Reported bruxism and pain from the temporomandibular joint were significantly higher in the 1942 cohort compared to the 1932 cohort, while reports of oral lesions and daytime dry mouth were lower. Women reported problems significantly more frequently in most of the domains investigated in both 2007 and 2017, that is, TMD, burning mouth, sensitive teeth, oral lesions, taste changes, daytime/night‐time dry mouth, except bad breath.

**Conclusions:**

TMD‐related symptoms increased while complaints from oral lesions and daytime mouth dryness decreased between 2007 and 2017. Temporal changes were otherwise few, but the findings underline the gender inequalities that exist, to the disadvantage of women. This must be considered when planning for clinical care/dental education to appropriately address the needs of older people.

## 1 INTRODUCTION

Gender inequalities in health‐related domains have for a long been of interest and many theories about their causes and consequences have been put forward. Women live longer than men but score lower in most domains related to health, functioning, and self‐reported well‐being, while older women suffer more from disability, loneliness, and depression compared to men (Carmel, 2019). Higher socioeconomic status, including education, income, and wealth, are strong determinants for enjoying good health in the elderly. The country in which one lives is also important and it has been reported that education and wealth are more important for health in women residing in Western and Southern Europe compared to those residing in Northern European countries (Uccheddu et al., 2019).

As regards gender differences related to oral health impairments in the elderly, women are more affected by poorer oral health‐related quality of life (Zusman et al., 2016), more frequent temporomandibular disorders (TMD), (Carlsson et al., 2014; Yadav et al., 2018) as well as having more intraoral problems (I. Johansson et al., 2016; Saintrain et al., 2018). Although the prevalence of TMD is known to increase from adolescence to middle age, there are few studies on its development in older ages and the available results are unclear (Bueno et al., 2018; Carlsson et al., 2014; Österberg & Carlsson, 2007). A recent review of incidence and sexual dimorphism of TMD in older adults concluded that most older adults have temporomandibular joint (TMJ) degeneration, affecting women more than men, peaks after child‐bearing age (45−64 years) and then gradually decreases with age but it is unclear how TMD affects older adults (Yadav et al., 2018). This notwithstanding, the question of how TMD prevalence is related to aging is not so clear and the often‐stated view that it decreases with age has been questioned (Carlsson et al., 2014).

Frequencies of orofacial symptoms in the elderly, and differences between genders, show few definitive conclusions. As regards the prevalence of burning mouth, a wide range from about 1% to ~20% (using varied diagnostic criteria) has been reported (Kohorst et al., 2015). In another study comparing middle‐aged and older patients with TMD and burning mouth, reported pain intensities were significantly higher in the older burning mouth group (65−84 years) while higher somatization and depression scores were found in the younger group (45−64 years). No such differences between the old and young groups were found among TMD patients but there was a significantly higher ratio of women in both the TMD and burning mouth patients (Honda et al., 2015). Overall, these findings confirm that burning mouth may constitute a significant problem among older patients and especially in women.

Life expectancy is increasing rapidly, while in parallel, the older population is generally healthier and they also increasingly retain their natural teeth into old age compared to their predecessor cohorts (A. K. Johansson, Omar et al., 2020). These developments are changing the nature of the demand for and utilization of dental services worldwide. Given these dynamics, it is important to document the contemporary oral health status among the elderly in order to obtain knowledge of possible trends for the benefit of both patients and dental professionals.

The objective of this study was to compare two cohorts of 75‐year‐olds examined in 2007 and 2017 with respect to reported TMD and orofacial symptoms, and with special reference to temporal changes and gender differences. The first hypothesis was that there are no differences between the two 75‐year‐old cohorts born 10 years apart and the second hypothesis was that women are generally more affected by oral health‐related problems than men.

## 2 MATERIALS AND METHODS

In 2007 and 2017, a questionnaire was sent to all 75‐year‐olds (born 1932 and 1942, respectively) residing in Örebro and Östergötland counties in Sweden. The response rates in 2007 and in 2017 were 71.9% (*n* = 3735; *N*_total_ = 5195) and 70.7% (*n* = 5091; *N*_total_ = 7204), respectively.

### 2.1 Questionnaire

The questionnaire comprised 56 questions in 2007 and 55 questions in 2017. Three questions about occupation that were included in 2007 were removed in the 2017 survey and substituted with two questions, one on type of residency and one on the ability to take themselves to the dentist. The questionnaire has been previously described and its methodological aspects discussed (Unell, 1999; Unell et al., 1997). In an early study in this series of investigations using the same methods and questionnaire, clinical examination was performed in 941 randomly selected subjects of the total sample in order to validate and quantify the responses regarding the reported number of remaining teeth and jaw opening capacity (Unell et al., 1997). There was good congruence between self‐reports and clinical registrations. Other oral conditions have not been validated. The questionnaire was split into six different categories which were related to: (i) social (viz. place of birth, marital status, education, residency), (ii) general health (e.g., physician visits, tobacco habits, medications), (iii) oral status (e.g., satisfaction with teeth, oral problems, oral hygiene habits, number of teeth), (iv) a series of attitude‐related questions (viz. oral function and appearance of teeth), and (v) experience and use of dental care services. A total of three mailing rounds of the questionnaire were performed. The first comprised information about the study, the questionnaire, and a prepaid reply envelope. The second was a reminder letter only and the third contained again a reminder, the questionnaire, and a prepaid reply envelope. In this paper, we focus on reported TMD and orofacial symptoms while other domains from the questionnaire have previously been reported (A. K. Johansson, Omar et al., 2020, A. Johansson et al., 2022).

### 2.2 Statistics

All statistical analyses were performed using the Statistical Package for the Social Sciences (SPSS; version 28) on an IBM Personal Computer. *χ* ^2^ tests were used to analyze differences between the 2007 and 2017 cohorts as well as between men and women. The significance level was set at *p* < .05.

### 2.3 Ethical considerations

The Ethics Committee in Uppsala, Sweden, approved the study (Dnr 2016/424). Informed consent was obtained from all the participants.

## 3 RESULTS

### 3.1 TMD‐related responses

Reported TMJ pain was significantly higher in 2017 than in 2007 (10.4% vs. 8.7%, *p* < .05). Women reported pain significantly more frequently than men in both the 2007 (10.0% vs. 7.2%) and 2017 (13.6% vs. 7.0%) cohorts (Table 1). The same applied to reported bruxism where there was an increase from 12.8% in 2007 to 16.6% in 2017 (*p* < .001). Here too women reported bruxism significantly more frequently than men in both 2007 (14.5% vs. 11.0%) and 2017 (18.1% vs. 15.1%). The increases in reported bruxism between the two examination points were similar for women (3.6%) and men (4.1%).

**Table 1:** Percentage distribution TMD‐related data in two cohorts of 75‐year‐old subjects, examined in 2007 and in 2017

	2007			2017			*p*
	Women	Men	Total	Women	Men	Total	
Pain from TMJ							
No problems	90.0	92.8	91.3	86.4	93.0	89.6	*, a
Some, a fair amount, or excessive problems	10.0	7.2	8.7	13.6	7.0	10.4	
TMJ sounds							
No problems	86.8	88.5	87.6	83.9	89.0	86.4	NS^b^
Some, a fair amount, or excessive problems	13.2	11.5	12.4	16.1	11.0	13.6	
Opening difficulties							
No problems	88.1	92.2	90.1	88.7	90.8	89.8	NS^c^
Some, a fair amount, or excessive problems	11.9	7.8	9.9	11.3	9.2	10.2	
Bruxism—self‐reported							
No problems	85.5	89.0	87.2	81.9	84.9	83.4	***, d
Some, a fair amount, or excessive problems	14.5	11.0	12.8	18.1	15.1	16.6	

*Note*: *p* denotes the comparison between the total figures in 2007 and 2017. Footnotes refer to gender differences for each cohort in 2007 and 2017.

Abbreviations: NS, not significant (Pearson's *χ* ^2^ test); TMD, temporomandibular disorders; TMJ,temporomandibular joint.

* .01 < *p* ≤ .05.

** .001 < *p* ≤ .01.

*** *p* ≤ .001.

^a^ 2007 cohort: **; 2017 cohort. ***

^b^ 2007 cohort: NS; 2017 cohort. ***

^c^ 2007 cohort: ***; 2017 cohort. *

^d^ 2007 cohort: **; 2017 cohort. **

There were no significant differences between 2007 and 2017 as regards reported TMJ sounds and mouth opening difficulties in the total samples. There were, however, gender differences regarding TMJ sounds in the 2017 cohort, and in both cohorts regarding opening difficulties with women reporting significantly more problems (Table 1). It should be noted that “a fair amount” or “excessive problems” constituted only a few percent in all four domains related to TMD.

### 3.2 Orofacial symptoms

For the total samples in both cohorts only “oral lesions” and “do you have dry mouth during the daytime?” showed significant changes over the examination time points, the former showing a decrease from 2007 (15.4%) to 2017 (12.2%) (*p* < .001) and similarly so for the latter (37.5% vs. 34.8%) (*p* < .05). Also, women reported problems significantly more frequently in both 2007 and 2017 for “burning mouth,” “oral lesions,” “sensitive teeth,” and dry mouth during daytime and night‐time (Table 2). Regarding “Change of taste,” women had significantly more difficulties than men in 2017 but not in 2007, and “bad breath” was significantly more common among men in both 2007 and 2017. “A fair amount” or “excessive problems” were relatively infrequently reported in most of the domains related to orofacial symptoms, except for problems of dry mouth where “often” was frequently reported and especially at night‐time and in both genders.

**Table 2:** Percentage distribution some orofacial symptoms in two cohorts of 75‐year‐old subjects, examined in 2007 and in 2017

	2007			2017			*p*
	Women	Men	Total	Women	Men	Total	
Burning mouth							
No problems	90.5	94.8	92.6	90.3	94.9	92.6	NS^a^
Some, a fair amount, or excessive problems	9.5	5.2	7.4	9.7	5.1	7.4	
Oral lesions							
No problems	83.3	86.0	84.6	86.1	89.6	87.8	***, b
Some, a fair amount, or excessive problems	16.7	14.0	15.4	13.9	10.4	12.2	
Sensitive teeth							
No problems	77.0	82.7	79.7	74.9	82.1	78.4	NS^c^
Some, a fair amount, or excessive problems	23.0	17.3	20.3	25.1	17.9	21.6	
Change of taste							
No problems	92.4	91.6	92.0	90.6	92.4	91.5	NS^d^
Some, a fair amount, or excessive problems	7.6	8.4	8.0	9.4	7.6	8.5	
Bad breath							
No problems	82.2	79.0	80.7	85.1	79.2	82.2	NS^e^
Some, a fair amount, or excessive problems	17.8	21.0	19.3	14.9	20.8	17.8	
Do you have dry mouth during daytime?							
No, rarely/never	56.4	69.3	62.5	59.5	71.3	65.2	*, f
Yes, often/sometimes	43.6	30.7	37.5	40.5	28.7	34.8	
Do you have dry mouth during night‐time?							
No, rarely/never	36.7	48.6	42.4	38.3	46.9	42.5	NS^g^
Yes, often/sometimes	63.3	51.4	57.6	61.7	53.1	57.5	

*Note*: *p* denotes the comparison between the total figures in 2007 and 2017. Footnotes refer to gender differences for each cohort in 2007 and 2017. NS, not significant (Pearson's *χ* ^2^ test).

* .01 < *p* ≤ .05.

** .001 < *p* ≤ .01.

*** *p* ≤ .001.

^a^ 2007 cohort: ***; 2017 cohort. ***

^b^ 2007 cohort: *; 2017 cohort. ***

^c^ 2007 cohort: ***; 2017 cohort. ***

^d^ 2007 cohort: NS; 2017 cohort. *

^e^ 2007 cohort: *; 2017 cohort. ***

^f^ 2007 cohort: ***; 2017 cohort. ***

^g^ 2007 cohort: ***; 2017 cohort. ***

## 4 DISCUSSION

In general terms, there have been declines in survey response rates over the past decades (Czajka & Beyler, 2016). A recent systematic review reported an average response rate of 65% for postal surveys related to the surgical field (Meyer et al., 2020). Elsewhere, the response rate in National Maternity Surveys in England decreased from 67% in 1995 to 29% in 2018 (Harrison et al., 2020). In another study conducted in 2016−2017 in which 17 rounds of 240 questionnaires related to sleep disturbances (total *n* = 4080) were mailed to randomly selected households living close to an airport, only 11.4% responded (Smith et al., 2019). In light of the foregoing, the response rate in the present study of over 70% for each of the two cohorts is clearly high, while both samples were fairly representative of the Swedish population of 75‐year‐olds as a whole, as previously discussed (A. K. Johansson, Omar et al., 2020).

Temporal changes between 2007 and 2017 in TMD and orofacial symptoms were relatively uncommon in the two cohorts. In another study, two cohorts of 70‐year‐old subjects were examined 8 years apart, and no significant difference between the two cohorts for prevalence of TMD symptoms was found (Österberg & Carlsson, 2007). In a previous report on the present samples, the 2017 cohorts reported much better general and oral health, the latter parameter is expressed as a significant improvement in chewing function, a stronger belief that they could keep their teeth throughout the whole of their lives, a substantially higher frequency of a full or almost full dentition, and a marked reduction in edentulousness and denture wearing (A. K. Johansson, Omar et al., 2020). In line with this, it might be expected that the same trend would be seen in relation to TMD. This was however not the case, with TMD‐related problems in fact increasing. This can be seen as support for the recent trend to emphasize psychological and behavioral factors as being more important than occlusal disturbances in the etiology of TMD.

Reported oral lesions and daytime dry mouth decreased, while the other examined symptoms showed no significant differences. The conclusion may thus be drawn that despite the global improvement in oral health among the elderly, there will still be a number of oral problems that need to be taken care of by the dental profession in the future aging population.

Not unexpectedly, women reported a significantly more frequent occurrence of TMD‐related problems than men in both 2007 and 2017 (except for TMJ sounds in 2007), which is in line with a systematic report on gender differences for TMD‐related problems (Bueno et al., 2018). On the other hand, and not so expectedly, approximately one‐third more (36%) of the women reported TMJ pain in 2017 compared to 2007 (representing an increase from 10.0% to 13.6%) whereas for men it remained at about the same level (7.0% vs. 7.2%). As regards self‐reported bruxism, both women and men showed substantial increases between the two same‐aged cohorts, with bruxism increasing in women from 14.5% to 18.1% and in men from 11.0% to 15.1%. This corresponds to a 25% for women and a 37% increase for men of self‐reported bruxism in 2017 compared to 2007. An increase in the prevalence of bruxism over time has also been shown in another study, (Egermark et al., 2001) and it has been suggested that this is related to the increased chronic stress in modern society (Wieckiewicz et al., 2014). It has however to be noted that self‐reported bruxism maybe is connected to bias and a definite diagnosis should also incorporate a clinical inspection in addition to polysomnography for sleep bruxism and electromyography for awake bruxism (Lobbezoo et al., 2018).

In a similarly‐designed study to the present one, of two cohorts of 50‐year‐olds examined 10 years apart in 1992 and 2002, there was a significantly higher prevalence of TMD‐related symptoms as well as impairments of other self‐reported health parameters in those born 10 years later (Unell et al., 2006). A recent systematic review had similar findings, with the authors referring to a changing epidemiology of TMD and the inference drawn that TMD prevalence was on the rise (Ryan et al., 2019). Our findings would suggest that this seems also to be the case for elderly Swedish women, but not for men.

Symptoms of burning mouth were reported almost twice as common in women compared to men in both 2007 and 2017 (~10% vs. ~5%). In another study and with stricter criteria applied, 0.17% of women were affected compared to only 0.04% of men among all age groups, although the highest prevalence was found in women aged 70−79 years, and to a lesser extent also in men of the same age group (Kohorst et al., 2015). In line with these results, the present findings confirm that burning mouth may constitute a significant problem among older patients and especially in women.

Reported oral lesions showed a decrease from 2007 (15.4%) to 2017 (12.2%). Taste changes were reported to be just under 10% in both cohorts, with women having significantly more of this condition in 2017. These findings show that these conditions are common in the elderly and, again, that women are more affected.

Bad breath was a commonly reported finding in both 2007 (19.3%) and 2017 (17.8%). A gender difference was also reported in the present study where men reported significantly more problems with bad breath than women, although to a lesser degree than in a Brazilian report (Nadanovsky et al., 2007). Nevertheless, bad breath is a significant problem in all age groups and continues to be so even among the elderly.

The prevalence of tooth hypersensitivity was higher among women and the results further indicated that the gender differences regarding sensitive teeth increased between 2007 and 2017 among 75‐year‐old Swedes (~5% difference in 2007 vs. ~7% difference in 2017). In a US study of 787 dental patients, aged 18−44, 45−64, and 65+ years, the overall prevalence of hypersensitivity was 12.3%, but in the 65+ years group, it was 6.1% (Cunha‐Cruz et al., 2013). In a multicenter, cross‐sectional French questionnaire survey of 2413 participants aged 18−65+ years, 42.2% reported tooth sensitivity during a 12‐month period while in the 65+ years group it was 35% (Blaizot et al., 2020). Thus, there was a wide range of tooth sensitivity prevalence which may have depended on diagnostic criteria applied, although there was a significant association between tooth sensitivity and female gender.

The prevalence of dry mouth was high both in the daytime (>30%) and night‐time (>50%), and with a striking gender difference. Mouth dryness was about 10% more common in women than in men both at night‐time and daytime and in both the 2007 and 2017 cohorts. The prevalence of reported dry mouth steadily increases from age 50 years (A. K. Johansson, Johansson et al., 2020). Although report of dry mouth is not necessarily a symptom of hyposalivation, a recent systematic review and meta‐analysis found a prevalence of over 30% in people aged over 60 years (Pina et al., 2020). A previous study emphasized the difference in the prevalence of daytime and night‐time dry mouth and suggested that “that day and night‐time xerostomia may be different conditions or at any rate have a somewhat different background” (A. K. Johansson et al., 2012). In this regard, salivary flow is lower at night which could be a part of the explanation for this finding (Dawes, 1972). The commonness of dry mouth and its overrepresentation in women further underlines the role that gender might play when assessing oral health in elderly people.

Two limitations with regard to the self‐administered questionnaire should be mentioned. First, even though concise explanatory notes accompanied the questionnaire to guide respondents in their interpretation and responses to questions/statements, the chances of less than precise responses being given cannot be excluded; nevertheless, the high response rate of more than 70% from all the 75‐year‐olds in the targeted counties may go some way to reducing this risk. Second, a known drawback with questionnaire surveys in general, is that they are at risk of conveying what respondents think they believe the interviewer wishes to hear, or indeed, in the absence of clear instructions and guidelines for answering questions, respondents may simply guess answers (Helminen et al., 2002). It would be reasonable to say that the accompanying explanatory notes in the questionnaire would have reduced such risks.

## 5 CONCLUSION

TMD‐related reported symptoms increased while complaints from oral lesions and daytime mouth dryness decreased between the two examination points in 2007 and 2017. Temporal changes were otherwise few and largely in line with the first hypothesis, but the findings of this study clearly underline the gender inequalities that exist in oral health‐related symptoms in the elderly population and confirmed the second hypothesis that women report poorer oral health than men. This has to be taken into account when planning for clinical care, as well as in the design of undergraduate and postgraduate dental education for appropriately addressing the needs of older people.

## AUTHOR CONTRIBUTIONS

*Investigation, project administration & data curation*: Josefin Sannevik and Berit Mastrovito. *Formal analysis*: Anders Johansson, Ridwaan Omar, Gunnar E. Carlsson, Josefin Sannevik, Berit Mastrovito, and Ann‐Katrin Johansson. *Writing – original draft preparation & writing – review and editing*: Anders Johansson, Ridwaan Omar, Gunnar E. Carlsson, Josefin Sannevik, Berit Mastrovito, and Ann‐Katrin Johansson.

## CONFLICT OF INTEREST

The authors declares no conflict of interest.

## Data Availability

The data set used and/or analyzed during the current study are available from the corresponding author on reasonable request.
